# A possible involvement of aberrant expression of the *FHIT* gene in the carcinogenesis of squamous cell carcinoma of the uterine cervix

**DOI:** 10.1038/sj.bjc.6690093

**Published:** 1999-02

**Authors:** S Nakagawa, H Yoshikawa, M Kimura, K Kawana, K Matsumoto, T Onda, N Kino, M Yamada, T Yasugi, Y Taketani

**Affiliations:** Department of Obstetrics and Gynecology, Faculty of Medicine, University of Tokyo, Bunkyo-ku, Tokyo, 113, Japan

**Keywords:** *FHIT* gene expression, cervical carcinoma, cervical intraepithelial neoplasia, squamous cell carcinoma, carcinogenesis, human papillomavirus

## Abstract

To investigate involvement of an aberrant expression of the *FHIT* (*f*ragile *h*istidine *t*riad) gene in the process of carcinogenesis and progression in cervical carcinoma, we examined its expression by the reverse transcriptase polymerase chain reaction (RT-PCR) and cDNA sequence method in 32 cervical invasive carcinomas (25 squamous cell carcinomas and seven adeno- or adenosquamous carcinomas) and 18 of its precursor lesions [four low-grade and 14 high-grade cervical intraepithelial neoplasias (CINs)]. We also examined a link between the occurrence of the aberrant expression and human papillomavirus (HPV). We detected the aberrant *FHIT* transcripts in 11 of 25 (44%) cervical invasive squamous cell carcinomas and in 5 of 14 (36%) high-grade CINs (CIN 2 or 3), whereas they were not found in seven non-squamous type and four low-grade CINs (CIN 1). The alteration patterns of the *FHIT* gene expression in high-grade CINs were virtually similar to those found in invasive carcinomas, such that the exons 5–7 were consistently deleted associated or unassociated with loss of the exon 4 and/or 8. The incidence of the aberrant expression was not related to the presence of HPV and its type. These data indicate that the aberrant expression of the *FHIT* gene is observed in precursor lesions of cervical carcinoma as well as invasive carcinomas, with its incidence not increasing with advance of clinical stage. Given the squamous cell type dominant expression, the aberrant expression may play a critical role in the generation of squamous cell carcinoma of the uterine cervix, but not the consequence of the progression of the cancer. © 1999 Cancer Research Campaign


*British Journal of Cancer* (1999) **79**(3/4), 589–594
© 1999 Cancer Research Campaign
Article no. bjoc.1998.0093

### A possible involvement of aberrant expression of the *FHIT* gene in the carcinogenesis of squamous cell carcinoma of the uterine cervix

**S Nakagawa, H Yoshikawa, M Kimura, K Kawana, K Matsumoto, T Onda, N Kino, M Yamada, T Yasugi and Y Taketani**

Department of Obstetrics and Gynecology, Faculty of Medicine, University of Tokyo, 7-3-1 Hongo, Bunkyo-ku, Tokyo 113, Japan

**Summary** To investigate involvement of an aberrant expression of the *FHIT* (*f*ragile *h*istidine *t*riad) gene in the process of carcinogenesis and progression in cervical carcinoma, we examined its expression by the reverse transcriptase polymerase chain reaction (RT-PCR) and cDNA sequence method in 32 cervical invasive carcinomas (25 squamous cell carcinomas and seven adeno- or adenosquamous carcinomas) and 18 of its precursor lesions [four low-grade and 14 high-grade cervical intraepithelial neoplasias (CINs)]. We also examined a link between the occurrence of the aberrant expression and human papillomavirus (HPV). We detected the aberrant *FHIT* transcripts in 11 of 25 (44%) cervical invasive squamous cell carcinomas and in 5 of 14 (36%) high-grade CINs (CIN 2 or 3), whereas they were not found in seven non-squamous type and four low-grade CINs (CIN 1). The alteration patterns of the *FHIT* gene expression in high-grade CINs were virtually similar to those found in invasive carcinomas, such that the exons 5–7 were consistently deleted associated or unassociated with loss of the exon 4 and/or 8. The incidence of the aberrant expression was not related to the presence of HPV and its type. These data indicate that the aberrant expression of the *FHIT* gene is observed in precursor lesions of cervical carcinoma as well as invasive carcinomas, with its incidence not increasing with advance of clinical stage. Given the squamous cell type dominant expression, the aberrant expression may play a critical role in the generation of squamous cell carcinoma of the uterine cervix, but not the consequence of the progression of the cancer.

**Keywords:** *FHIT* gene expression; cervical carcinoma; cervical intraepithelial neoplasia; squamous cell carcinoma; carcinogenesis; human papillomavirus

It has been postulated that inactivation of products of both *p53* and retinoblastoma susceptibility (*Rb*) genes by binding with E6 and E7 proteins of oncogenic human papillomaviruses (HPVs), respectively, may be possible pathogenic factors in the development of cervical carcinoma (Munger et al, 1989; Scheffner et al, 1990; Werness et al, 1990; Huibregtse et al, 1991). However, the mutation frequency of these genes in this malignancy is much lower than other malignancies (Choo and Chong, 1993). Besides, based on the clinical evidence, HPV infection alone may be unlikely to explain the mechanism of malignant transformation of cervical epithelial cells.

Abnormal transcripts of the fragile histidine triad (*FHIT*) gene located at 3p14.2 have been identified in a variety of human carcinomas (Mao et al, 1996; Ohta et al, 1996; Sozzi et al, 1996; Fong et al, 1997; Gemma et al, 1997; Larson et al, 1997). The 3p14.2 lies within the short arm of chromosome 3 (3p13–21.1), where allelic deletions have been frequently observed in cervical carcinoma (Wilke et al, 1996; Larson et al, 1997; Wistuba et al, 1997). Integration of HPV DNA has been identified at a fragile site (*FRA*3B) within the *FHIT* locus in cervical cancer (Wilke et al, 1996). A recent study showed that alterations of the *FHIT* gene expression were more frequently found in cervical cancer cell lines compared with ovarian and endometrial cancer cell lines (Hendricks et al, 1997). In connection with this finding, the aberrant *FHIT* transcripts were found in 17 of 25 (68%) primary cervical carcinomas (Greenspan et al, 1997). Furthermore, loss of heterozygosity of the locus containing the *FHIT* gene was frequently found in cervical intraepithelial neoplasias (CINs) adjacent to invasive cervical carcinomas (Wistuba et al, 1997).

To investigate a possible involvement of the aberrant expression of the *FHIT* gene in the carcinogenesis of cervical carcinoma, we examined the *FHIT* gene expression by the reverse transcriptase polymerase chain reaction (RT-PCR) and cDNA sequence methods in cervical carcinomas with different stages, and its precursor lesions (low-grade and high-grade CINs) as well. We also looked at the relation between the occurrence of the aberrant *FHIT* gene expression and the presence of HPV and its type.

*Received 29 January 1998*
*Revised 23 April 1998*
*Accepted 6 May 1998*

*Correspondence to:* H Yoshikawa

### MATERIALS AND METHODS

### Tissues samples

The tissue specimens were obtained from 32 cervical carcinoma and 18 CIN patients who were treated at the University of Tokyo Hospital. Fresh biopsied specimens of cervical neoplastic tissue before surgery or radiotherapy were obtained from patients who gave informed consent to use them for research purposes. Corresponding control tissue samples of the cervix and the vagina were obtained only from the women who gave informed consent (35 out of 50 cases). The biopsied tissue samples were snap frozen in liquid nitrogen or frozen at –80°C immediately after excision. Cryostat sections were cut for haematoxylin and eosin staining to confirm the spread of the lesion in cancer or CIN tissue samples, and non-neoplastic tissues were trimmed off from the frozen tissues. Tissues in which neoplastic cells comprised more than 70% of total cells were used for RNA and DNA extraction. Regarding control tissue samples, the tissues over 70% of which were made up of epithelial cells were used for RNA extraction by trimming of stromal tissue. Clinical information was obtained by chart review. Of the 32 invasive carcinoma patients, 25 had squamous cell carcinoma (SSC), six had adenocarcinoma (AC) and one had adenosquamous carcinoma (ASC). Of the 18 patients with CIN, four had low-grade CIN (CIN 1) and 14 had high-grade CIN (CIN 2 or 3). The average age of invasive carcinoma cases was 52 years with a range from 26 to 82 years, and that of CIN cases was 41 years with a range from 19 to 78 years. Staging of invasive carcinoma cases, based on the International Federation of Gynecology and Obstetrics (FIGO) criteria was as follows: 15, stage I; eight, stage II; six, stage III; and three, stage IV (Table 1).

### RNA isolation and reverse transcription (RT)

Total RNA was isolated using the RNeasy Mini Kit (Qiagen, Chatsworth, CA, USA). The mRNA was isolated from the total RNA by the Oligotex-dT Super(TaKaRa Biomedicals, Japan). Reverse transcription was performed in a 10-μl volume of standard buffer (Gibco, Gaithersburg, MD, USA), 500 μM dNTPs, 200 μM oligo(dT) primer, 20 U of Human Placenta RNasin, and 200 U of M-MLV Reverse Transcriptase (Gibco). The mRNA preheated at 65°C for 5 min was used for RT reaction at 37°C for 60 min, and the reaction was stopped by inactivating the enzyme at 95°C for 3 min.

### RT PCR and cDNA sequencing method

We used the nested PCR method reported by Ohta et al, 1996) to analyse the *FHIT* gene expression. The first PCR was performed in a volume of 25 μl containing 0.8 μM of primers of 5U2(5′-ATCCTGGAAGCTTTGAAGCTCA-3′) and 3D2(5′-TCACTG-GTTGAATACAGGA-3′), 50 μM of each dNTP, 1 × PCR buffer, and 1 U of the Ampli *Taq* Gold (Perkin-Elmer Applied Biosystems). The PCR reaction was performed for 25 cycles as reported by Ohta et al (1996) in a Perkin-Elmer thermal cycler (model 2400), with the exception that the hot start (95°C for 7 min) was added before the thermal cycling. One microlitre of 20-fold-diluted amplified product was subjected to a second PCR amplification with nested primers 5U1 (5′-TCCGTAGTGCTATC-TACATC-3′) and 3D1 (5′-CATGCTGATTCAGTTCCTCTTGG-3′) for 30 cycles. To analyse the *FHIT* gene expression with accuracy, we used different sets of primers MUR5 and RP2 (Hendricks et al, 1997). The PCR products were electrophoresed on 2% agarose gels (NuSieve, FMC Bioproducts, Rockland, ME, USA). Bands were cut from agarose gels, and DNA was extracted using the Suprec-01 (TaKaRa). DNA was sequenced directly using primers 5U1 and 3D1, by the *Taq* DyeDeoxy terminator cycle sequencing reaction for sequence analysis on the Applied Biosystems model 373A.

### Detection and typing of HPV

The PCR for the L1 region was carried out by using the consensus L1 primers L1C1 (5′-CGTAAACGTTTTCCCTATTTTTTT-3′) (1 μM), L1C2 (5′-TACCCTAAATACTCTGTATTG-3′) (0.5 μM), and L1C2M (5′-TACCCTAAATACCCTATATTG-3′) (0.5 μM) for the PCR. Other conditions of PCR were as described elsewhere (Yoshikawa et al, 1991). Briefly, the PCR protocol was 40 cycles, and each cycle consisted of 1.5 min denaturation (95°C), 1.5 min annealing (48°C) and 2 min extension (70°C). One-tenth of the reaction was electrophoresed on a 4% agarose gel and stained with ethidium bromide. HPV types were identified on the basis of the restriction fragment length polymorphisms (RFLP). The PCR-based assay (L1-PCR) can type at least 26 registered genital HPVs such as type 6, 11, 16, 18, 30, 31, 33, 34, 35, 39, 42, 43, 44, 45, 51, 52, 53, 54, 55, 56, 58, 59, 61, 66, 68 and 70 (Yoshikawa et al, 1991). This PCR system can detect 0.01 pg of HPV DNA for all these types except for HPV DNA of types 34, 42 and 55, of which 0.1 μg DNA is minimally needed for detection. We used β-actin gene amplification to rule out false-negative results.

### Statistical analysis

The statistical correlation between the *FHIT* gene expression and clinical characteristics were analysed by the $\chi^2$ method, global $\chi^2$ test and the Fisher exact test. The correlation between the *FHIT* gene expression and age of the patient were analysed by the ANOVA approach. All statistical analyses were carried out with StatView 4.11, with statistical significance set at 5%.

### RESULTS

### Alteration of the *FHIT* gene expression in invasive cervical carcinomas

All the 32 cervical carcinomas had detectable transcripts of expected and/or an aberrant size of the *FHIT* gene in the RT-PCR assays. Each aberrant *FHIT* band was observed reproducibly by at least two different RT-PCR analyses. In all the corresponding normal cervical or vaginal tissue samples, only the *FHIT* transcript with expected size was identified. Aberrant transcripts of the *FHIT* gene were detected in 11 out of 32 (34%) carcinomas. Among these 11 carcinomas, three had only one band of the aberrantly truncated *FHIT* transcript without the transcript of expected size (Figure 1). The remaining eight carcinomas had the transcript of expected size in addition to the aberrantly truncated *FHIT* transcripts (Figure 1). Sequence analysis of the aberrant *FHIT* cDNA revealed that all the 11 carcinomas had, at least, a deletion of exon 5–7 in common (a deletion of exon 4–7 in five, exon 5–7 in five, and exon 4–8 in one, Figure 2). One out of the five cases which lacked exon 5–7 had also an insertion of a 36-bp sequence between exons 4 and 8 (Figure 2). Two cases which had the aberrant transcripts with a deletion of exon 4–7 showed a T to C silent mutation at nt 294 (Figure 2). All of the *FHIT* cDNA of expected size detected in the eight cases contained the transcript of wt sequence (707 bp) and/or the alternatively spliced transcript with 11 bp deletion (nt 812–822) in exon 10 (696 bp, termed *FHITβ*; Mao et al, 1996).

### Alteration of the *FHIT* gene expression in CINs

We examined the aberrant *FHIT* gene expression in 18 CIN tissues. The aberrant *FHIT* transcript was not found in four cases with low-grade CIN (CIN1), whereas it was identified in 5 of the 14 cases with high-grade CIN (CIN2 or 3, Figure 1); a deletion of exon 4–7 in one, a deletion of exon 4–8 in two, a deletion of exon 4 in one, and a deletion of exon 5–7 and exon 4–8 in one (Figure 2). All of the high-grade CINs had the transcripts of the expected

© Cancer Research Campaign 1999

**Table 1** The *FHIT* gene expression, HPV type, and clinical data

**CIN**

| No. | Age | CIN grade | HPV type | *FHIT* gene expression | |
|---|---|---|---|---|---|
| | | | | Normal control | Tumour tissue |
| 1 | 78 | CIN 1 | 16 | N[a] | N |
| 2 | 19 | CIN 1 | 56 | | N |
| 3 | 22 | CIN 1 | 58 | | N |
| 4 | 21 | CIN 1 | 59 | N | N |
| 5 | 32 | CIN 2 | 16 | N | N and Ab[b] (Del. [c]exon 5–7, 4–8) |
| 6 | 40 | CIN 2 | 33 | | N |
| 7 | 43 | CIN 2 | 52 | N | N and Ab (Del. exon 4) |
| 8 | 32 | CIN 3 | 16 | N | N and Ab (Del. exon 4–8) |
| 9 | 34 | CIN 3 | 16 | | N |
| 10 | 40 | CIN 3 | 16 | N | N |
| 11 | 45 | CIN 3 | 16 | | N |
| 12 | 46 | CIN 3 | 16 | N | N |
| 13 | 56 | CIN 3 | 16 | | N |
| 14 | 43 | CIN 3 | 31 | N | N and Ab (Del. exon 4–7) |
| 15 | 66 | CIN 3 | 35 | N | N |
| 16 | 26 | CIN 3 | 51 | N | N |
| 17 | 53 | CIN 3 | 52 | N | N and Ab (Del. exon 4–8) |
| 18 | 33 | CIN 3 | 58 | N | N |

**Invasive carcinoma**

| No. | Age | Histology | Stage | HPV type | *FHIT* gene expression | |
|---|---|---|---|---|---|---|
| | | | | | Normal control | Tumour tissue |
| 1 | 46 | SCC | Ia | 52 | N | N and Ab (Del. exon 4–7, Nt.[d] 294 T→C) |
| 2 | 26 | SCC[e] | Ib | 16 | N | Ab (Del. exon 5–7) |
| 3 | 33 | AC[f] | Ib | 16 | N | N |
| 4 | 54 | SCC | Ib | 16 | N | N and Ab (Del. exon 4–7) |
| 5 | 58 | SCC | Ib | 16 | N | N and Ab (Del. exon 4–8) |
| 6 | 75 | AC | Ib | 16 | | N |
| 7 | 29 | SCC | Ib | 18 | N | N and Ab (Del. exon 4–7) |
| 8 | 32 | SCC | Ib | 18 | N | N |
| 9 | 41 | ASC[g] | Ib | 18 | | N |
| 10 | 43 | SCC | Ib | 18 | N | N and Ab (Del. exon 5–7, insertion 36 bp) |
| 11 | 50 | SCC | Ib | 18 | N | N |
| 12 | 65 | SCC | Ib | 18 | | N |
| 13 | 36 | AC | Ib | Negative | N | N |
| 14 | 47 | SCC | Ib | Negative | N | Ab (Del. exon 5–7) |
| 15 | 65 | SCC | Ib | Negative | N | N and Ab (Del. exon 5–7) |
| 16 | 52 | AC | IIa | 16 | | N |
| 17 | 39 | SCC | IIb | 16 | N | N |
| 18 | 47 | SCC | IIb | 16 | N | N |
| 19 | 64 | SCC | IIb | 16 | N | N |
| 20 | 39 | SCC | IIb | 18 | | N |
| 21 | 82 | SCC | IIb | 31 | N | N and Ab (Del. exon 4–7, Nt. 294 T → C) |
| 22 | 57 | SCC | IIb | 35 | | N |
| 23 | 39 | SCC | IIb | 58 | N | N |
| 24 | 35 | SCC | IIIb | 16 | N | Ab (Del. exon 5–7) |
| 25 | 45 | SCC | IIIb | 16 | N | N |
| 26 | 51 | SCC | IIIb | 31 | N | N and Ab (Del. exon 4–7) |
| 27 | 68 | SCC | IIIb | 31 | | N |
| 28 | 69 | SCC | IIIb | 33 | N | N |
| 29 | 69 | AC | IIIb | 52 | N | N |
| 30 | 79 | SCC | IVa | 16 | N | N |
| 31 | 61 | AC | IVa | Negative | | N |
| 32 | 64 | SCC | IVa | Negative | | N |

[a]N, normal; [b]Ab, aberrant; [c]Del, deletion; [d]Nt, nucleotide; [e]SCC, squamous cell carcinoma; [f]AC, adenocarcinoma; [g]ASC, adenosquamous carcinoma.

© Cancer Research Campaign 1999

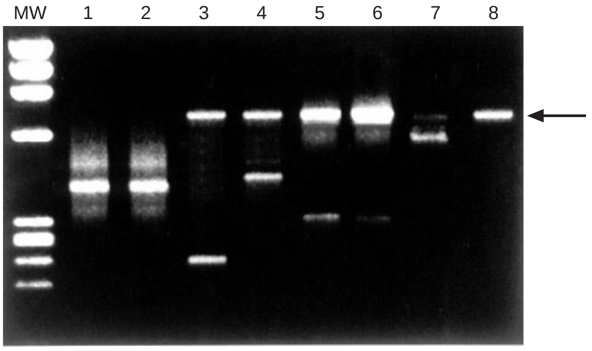

**Figure 1** Aberrant transcripts of the *FHIT* gene in cervical invasive carcinomas and CINs. MW, ϕ×174-HaeIII fragment. Lanes 1–5, RT-PCR analysis of cervical invasive carcinomas (lanes 1 and 2, aberrant transcript only; lanes 3–5, aberrant and normal transcripts). Lanes 6 and 7, RT-PCR analysis of high-grade CINs (aberrant and normal transcripts). Lane 8, RT-PCR analysis of normal cervical tissue (normal transcript). Arrowhead indicates the normal-sized (707 bp) transcript size in addition to the aberrant transcripts. Like invasive carcinomas, the transcripts of the expected size had the transcript of wt sequence (707 bp) and/or the alternatively spliced transcript with 11 bp deletion (nt 812–822) in exon 10 (696 bp).

### The presence of HPV and its type in invasive cervical carcinomas and CINs

HPV DNA was detected in 27 out of 32 invasive cervical carcinomas and 18 out of 18 in CINs. The type of HPV in 32 invasive carcinomas was as follows: type 16 in 12; type 18 in seven; type 31 in three; type 33 in one; type 35 in one; type 52 in two; type 58 in one; and HPV negative in five cases. Type of HPV in the CINs was as follows: type 16 in eight; type 31 in one; type 33 in one; type 35 in one; type 51 in one; type 52 in two; type 56 in one; type 58 in two; and type 59 in one case. All HPV types detected in this study were cervical cancer-associated HPV, which were subdivided into high-risk HPV (types 16 and 18) and intermediate-risk HPV (the other types). The presence of HPV and its type in each invasive carcinoma and CIN are shown in Table 1. We examined the incidence of the aberrant *FHIT* gene expression in relation to the HPV type. In invasive carcinomas, the incidence of aberrant *FHIT* gene was 33% (4 out of 12) in HPV-16-positive cases, 29% (two out of seven) in HPV-18-positive cases, 38% (three out of eight) in HPV other than HPV-16- and HPV-18-positive cases and 40% (two out of five) in HPV-negative cases. In CINs, the incidence was 25% (two out of eight) in HPV-16-positive cases and 30% (three out of ten) in HPV other than HPV-16- and HPV-18-positive cases. There was no significant relation between the incidence of the aberrant *FHIT* gene expression and HPV type in both invasive carcinomas and CINs.

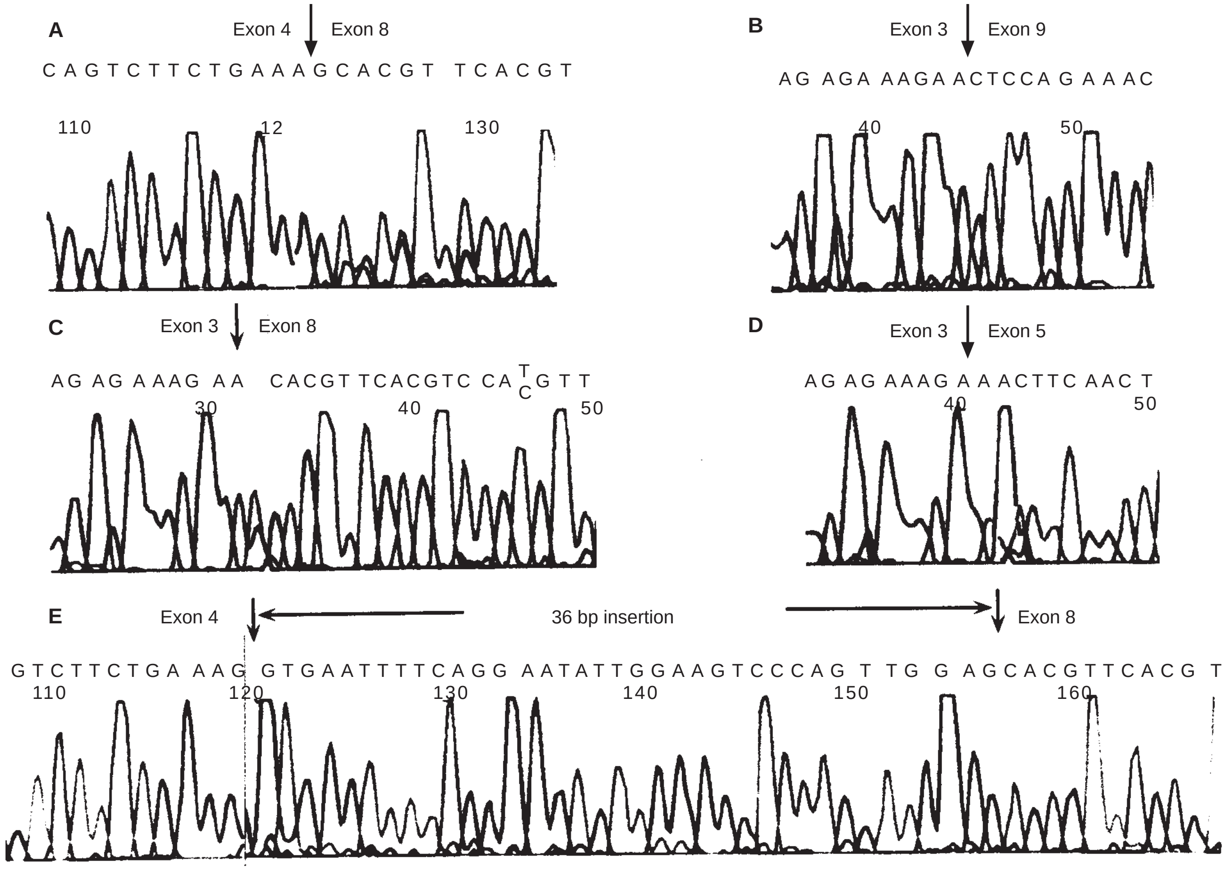

**Figure 2** Sequence analysis of the aberrant *FHIT* transcripts of cervical invasive carcinomas (**A**, **B**, **C** and **E**) and high-grade CIN (**D**)

© Cancer Research Campaign 1999

### Relation of the aberrant *FHIT* gene expression with clinical characteristics in invasive cervical carcinomas

We analysed the relation of the aberrant *FHIT* gene expression with clinical characteristics such as histology, age at diagnosis and clinical stage (Table 1).

The aberrant *FHIT* gene expression was detected more frequently in invasive squamous cell carcinomas than in invasive non-squamous carcinomas [44% (11 out of 25) vs 0% (zero out of seven), $P = 0.03$, Fisher exact test].

The mean age of the patients with the aberrant *FHIT* gene expression (49 years) was slightly younger than that of the patients with normal *FHIT* gene expression (54 years), though the difference was not significant ($P = 0.40$).

We compared the incidence of the aberrant *FHIT* gene expression according to clinical stage of invasive carcinoma. The incidence of the aberrant *FHIT* gene expression in each stage was as follows: 53% (8 out of 15) in stage I; 13% (one out of eight) in stage II; 33% (two out of six) in stage III; and 0% (zero out of three) in stage IV. Although a statistically higher incidence of the aberrant *FHIT* gene expression was noticed in stage I cases ($P = 0.01$, Fisher exact 2×2 analysis) compared with that in stage II or more advanced (stage II–IV), there is no noticeable relation between the incidence and clinical stage by global $\chi^2$ test ($P = 0.13$), suggesting that there was no tendency of an increase in the incidence of the aberrant *FHIT* gene expression with advance of clinical stage.

### DISCUSSION

It is still open as to whether the aberrant *FHIT* gene expression occurs before development of invasive cervical carcinoma. If this is the case, the aberrant expression of the gene may play an important role in the carcinogenesis of cervical cancer. Here, we found the aberrant *FHIT* transcripts in 5 out of 14 (35.7%) women with high-grade CIN, a putative precursor lesion of invasive cervical carcinoma of squamous cell type. The alteration pattern of the aberrant transcript in high-grade CINs was essentially similar to that in invasive carcinomas, with the exception of one CIN case which had a deletion of exon 4.

A recent report (Wistuba et al, 1997) showed a frequent occurrence is a deletion of chromosome 3p containing the *FHIT* locus in high-grade CINs adjacent to invasive carcinomas. In the present study, frequent alteration of *FHIT* gene expression was observed in isolated high-grade CIN unassociated with invasive lesion. Additionally, the incidence of the alteration did not increase with the advance of clinical stage of carcinomas (Table 1). These data suggest that the alteration of the *FHIT* gene may be an early genetic event during the generation of cervical carcinoma, but not the consequence of cancer progression.

The RT-PCR and cDNA sequencing revealed that deletions of the *FHIT* gene were more frequent than a point mutation or rearrangement in invasive cervical carcinoma and CIN. In this study, the exons 5–7 were invariably deleted with or without loss of exon 4 and/or 8 in the aberrant transcripts of the *FHIT* gene detected. These results are consistent with those found in other various carcinomas (Mao et al, 1996; Ohta et al, 1996; Sozzi et al, 1996; Fong et al, 1997; Gemma et al, 1997; Larson et al, 1997) and in cervical carcinoma cell lines (Greenspan et al, 1997; Hendricks et al, 1997). However, this report is first to analyse precisely the deletion of the *FHIT* cDNA in primary cervical carcinoma. Considering the fact that the predicted translation initiation codon resides in exon 5, the aberrant *FHIT* transcript may fail to generate any protein product. In 8 of 11 invasive carcinomas with aberrant *FHIT* transcript, the normal-sized transcript was detected as well. The coexistence of the aberrant *FHIT* transcript and the normal-sized transcript is in keeping with previous reports which investigated various types of cancer cell lines and primary carcinomas (Mao et al, 1996; Ohta et al, 1996; Sozzi et al, 1996; Fong et al, 1997; Gemma et al, 1997; Larson et al, 1997). Though we used the tissue specimens in which neoplastic cells constituted more than 70% of the total cells for RNA extraction, it is impossible to exclude any normal stromal cells and lymphocytes from primary tissue specimens. Thus, a possibility that the normal-sized transcript is derived not from neoplastic cells but from contaminating normal cells cannot be discounted.

Several studies indicated an inverse relationship between *p53* mutation and HPV positivity in cervical carcinomas (Crook et al, 1991; Crook and Vousden, 1992). It was further shown that *p53* mutations were confined to the HPV-negative cell lines derived from cervical carcinomas, although recent studies have questioned the relation between *p53* and HPV (Fujita et al, 1992; Choo and Chong, 1993; Helland et al, 1993; Miwa et al, 1995). Regarding a link between HPV and the *FHIT* gene, integration of HPV DNA has been identified at a fragile region (FRA3B) within the *FHIT* gene in cervical carcinoma (Wilke et al, 1995). However, in view of the present result showing that there seems to be no association of the incidence of the aberrant *FHIT* gene expression with the presence of HPV or its type in both invasive carcinoma and CIN, the aberrant *FHIT* gene expression could participate in the carcinogenesis presumably in a manner unrelated to HPV infection.

The incidence of the aberrant *FHIT* gene expression varies according to histopathological subtypes in certain tumours. For instance, small-cell lung cancers show more frequent alteration of the *FHIT* gene expression than other types of lung cancer (Sozzi et al, 1996). In this study, the aberrant *FHIT* transcripts were detected only in cervical invasive carcinomas with squamous cell type. It was reported that the alteration of the *FHIT* gene expression was more frequent in cervical cancer cell lines (five out of eight) compared with cell lines derived from ovarian (2 out of 14) or endometrial carcinomas (zero out of four), which are predominantly adenocarcinoma (Hendricks et al, 1997). Taken together, these data suggest that *FHIT* alterations may be more closely related to carcinogenesis of squamous cell carcinoma than to that of adenocarcinoma in gynaecological malignancies.

In conclusion, we demonstrated that the aberrant expression of the *FHIT* gene was found in CIN as well as invasive cervical carcinoma, with its incidence being unchanged during progressional process. Given the squamous cell carcinoma dominant occurrence, the aberrant expression of the *FHIT* gene may play a critical role in the generation of squamous cell carcinoma arising from the uterine cervix.

### ACKNOWLEDGEMENTS

We are grateful to Dr Tadahito Kanda (National Institute of Infectious Diseases, Division of Molecular Genetics) for providing scientific advice. This work was supported by a cancer research grant from the Ministry of Education, Science, and Culture of Japan (A03-07272105).

### REFERENCES

Choo K-B and Chong KY (1993) Absence of mutation in the p53 and the retinoblastoma susceptibility genes in primary cervical carcinomas. *Virology* **193**: 1042–1046

© Cancer Research Campaign 1999

Crook T and Vousden KH (1992) Properties of p53 mutations detected in primary and secondary cervical cancers suggest mechanisms of metastasis and involvement of environmental carcinogens. *EMBO J* **11**: 3935–3940

Crook T, Wrede D and Vousden KH (1991) p53 point mutation in HPV negative human cervical carcinoma cell lines. *Oncogene* **6**: 873–875

Fong KM, Biesterveld EJ, Virmani A, Wistuba I, Sekido Y, Bader SA, Ahmadian M, Ong ST, Rassool FV, Zimmerman PV, Giaccone G, Gazdar AF and Minna JD (1997) *FHIT* and *FRA*3B 3p14.2 allele loss are common in lung cancer and preneoplastic bronchial lesions and are associated with cancer-related *FHIT* cDNA splicing aberrations. *Cancer Res* **57**: 2256–2267

Fujita M, Inoue M, Tanizawa O, Iwamoto S and Enomoto T (1992) Alterations of the *p53* gene in human primary cervical carcinoma with and without human papillomavirus infection. *Cancer Res* **52**: 5323–5328

Gemma A, Hagiwara K, Ke Y, Burke LM, Khan MA, Nagashima M, Bennett WP and Harris CC (1997) *FHIT* mutations in human primary gastric cancer. *Cancer Res* **57**: 1435–1437

Greenspan DL, Connolly DC, Wu R, Lei RY, Vogelstein JTC, Kim Y-T, Mok JE, Munoz N, Bosch FX, Shah K and Cho KR (1997) Loss of *FHIT* expression in cervical carcinoma cell lines and primary tumors. *Cancer Res* **57**: 4692–4698

Helland A, Holm R, Kristensen G, Laern J, Kaern J, Karlsen F, Trope C, Nesland JM and Borresen AL (1993) Genetic alterations of the TP53 gene, p53 protein expression and HPV infection in primary cervical carcinomas. *J Pathol* **171**: 105–114

Hendricks DT, Taylor R, Reed M and Birrer MJ (1997) *FHIT* gene expression in human ovarian, endometrial, and cervical cancer cell lines. *Cancer Res* **57**: 2112–2115

Huibregtse JM, Scheffner M and Howley PM (1991) A cellular protein mediates association of p53 with the E6 oncoprotein of human papillomavirus types 16 or 18. *EMBO J* **10**: 4129–4135

Larson AA, Kern S, Curtiss S, Gordon R, Cavenee WK and Hampton GM (1997) High resolution analysis of chromosome 3p alterations in cervical carcinoma. *Cancer Res* **57**: 4082–4090

Mao L, Fan YH, Lotan R and Hong WK (1996) Frequent abnormalities of *FHIT*, a candidate tumor suppressor gene, in head and neck cancer cell lines. *Cancer Res* **57**: 5128–5131

Miwa K, Miyamoto S, Imamura T, Nishida M, Yoshikawa Y, Nagata Y and Wake N (1995) The role of p53 inactivation in human cervical cell carcinoma development. *Br J Cancer* **71**: 219–226

Munger K, Werness BA, Dyson N, Phelps WC, Harlow E and Howley PM (1989) Complex formation of human papillomavirus E7 proteins with the retinoblastoma tumor suppressor gene product. *EMBO J* **8**: 4099–4105

Ohta M, Inoue H, Cotticelli MG, Kastury K, Baffa R, Palazzo J, Siprashvili Z, Mori M, McCue P, Druck T, Croce CM and Hebner K (1996) The *FHIT* gene, spanning the chromosome 3p14.2 fragile site and renal carcinoma-associated t(3;8) breakpoint, is abnormal in digestive tract cancers. *Cell* **84**: 587–597

Scheffner M, Werness BA, Huibregtse JM, Levine AJ and Howley PM (1990) The E6 oncoprotein encoded by human papillomavirus types 16 and 18 promotes the degradation of p53. *Cell* **63**: 1129–1136

Sozzi G, Veronese ML, Negrini M, Baffa R, Cotticelli MG, Inoue H, Tornielli S, Pilotti S, De Gregorio L, Pastorino U, Pierotti Ma, Ohta M, Huebner K and Croce CM (1996) The *FHIT* gene 3p14.2 is abnormal in lung cancer. *Cell* **85**: 17–26

Werness BA, Levine AJ and Howley PM (1990) Association of human papillomavirus types 16 and 18 E6 proteins with p53. *Science* **248**: 76–79

Wilke CM, Hall BK, Hoge A, Paradee W, Smith DI and Glover TW (1996) FRA3B extends over a broad region and contains a spontaneous HPV16 integration site: direct evidence for the coincidence of viral integration sites and fragile sites. *Hum Mol Genet* **5**: 187–195

Wistuba I, Montellano FD, Milchgrub S, Virmani AK, Behrens C, Chen H, Ahmadian M, Nowak JA, Muller C, Minna JD and Gazdar AF (1997) Deletions of chromosome 3p are frequent and early events in the pathogenesis of uterine cervical carcinoma. *Cancer Res* **57**: 3154–3158

Yoshikawa H, Kawana T, Kitagawa K, Mizuno M, Yoshikura H and Iwamoto A (1991) Detection and typing of multiple genital human papillomaviruses by DNA amplification with consensus primers. *Jpn J Cancer Res* **82**: 524–531

*© Cancer Research Campaign 1999*
